# Supplementary material for: Derivatization of Bufadienolides at Carbon-3 of the Steroid Core and Their Consequences for the Interaction with Na+,K+-ATPase
Source: Int J Mol Sci. 2025 Nov 14;26(22):11027. doi: 10.3390/ijms262211027 (PMC12652054; doi:10.3390/ijms262211027)
Supplement: Supplementary file 1 [file ijms-26-11027-s001.zip › ijms-3981046-supplementary.pdf]

Supplementary Information

Derivatization of Bufadienolides at Carbon-3 of the Steroid Core and Their Consequences for the Interaction with Na^+,K^+-ATPase

Lucy Kate Ladefoged [1,2,†], Birgit Schiøtt [2,‡] and Natalya U. Fedosova [1,*]

1 Department of Biomedicine, Aarhus University, Høegh-Guldbergsgade 10, 8000 Aarhus C, Denmark
2 Department of Chemistry, Aarhus University, Langelandsgade 140, 8000 Aarhus C, Denmark
* Correspondence: nf@biomed.au.dk
† Current affiliation: Raven Biosciences, Åbogade 15, 8200 Aarhus N, Denmark.
‡ Current affiliation: Faculty of Natural Sciences, Aarhus University, Ny Munkegade 116, 8000 Aarhus C, Denmark.

Contents:

Supporting Figure S1. Affinity of cardiotonic steroids in Histidine buffer.
Supporting Figure S2. Chemical structures of CTS.
Supporting Figure S3. Starting conformations for MD simulations.
Supporting Figure S4. Overview of possible intermolecular hydrogen bonds.
Supporting Figure S5. Ligand movement along the z-axis.
Supporting Figure S6. Ligand tilting.
Supporting Figure S7. Estimated solubility and lipophilicity.
Supporting Figure S8. Dynamic range of sugar moieties in digoxin.
Supportigg Figure S9. RMSD progression of simulated systems.
Supporting Data File S1. Digoxin force field parameters.
Supporting Data File S2. Bufalin-N-glucose force field parameters.

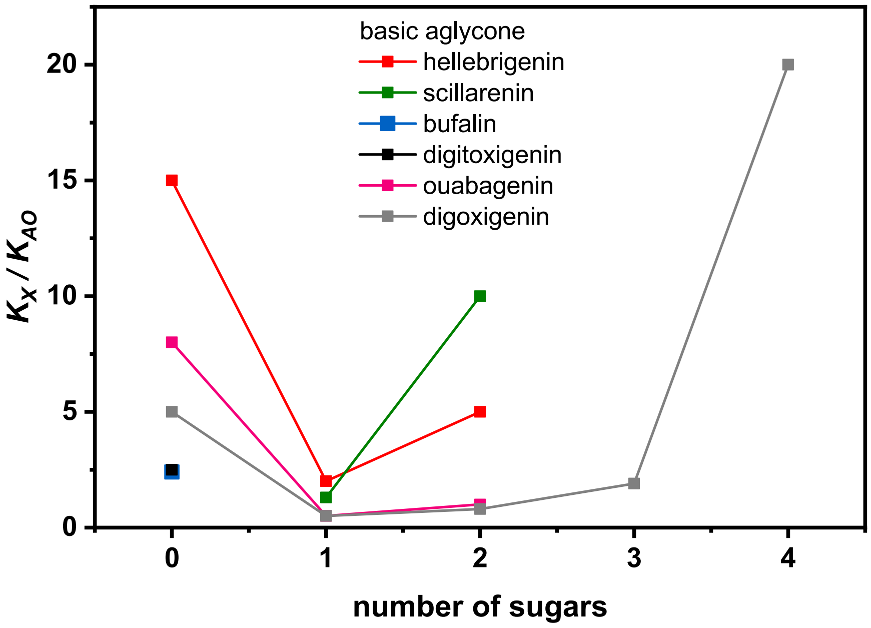

Supporting Figure S1. The ratio of the dissociation constants of CTS (K_X) to that of anthroylouabain (AO) (K_{AO}) as a function of a degree of glycosylation. Experiments were performed in His buffer and in the absence of K^+. All compounds are O-glycosylated.

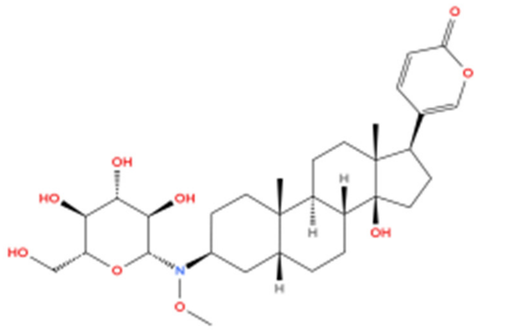

Bufalin-N-glucose

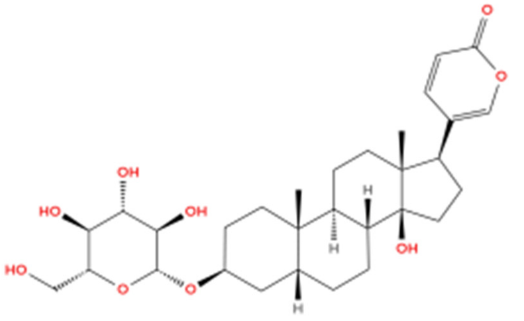

Bufalin-O-glucose

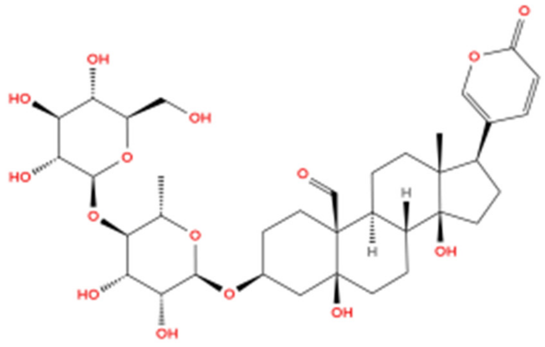

Hellebrin

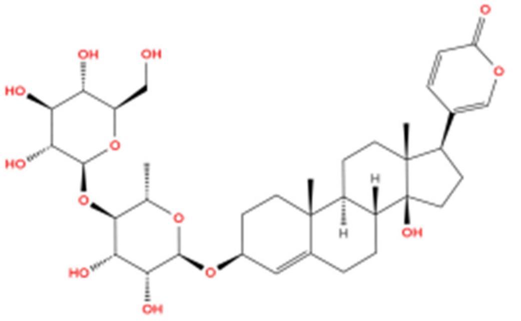

Scillaren A

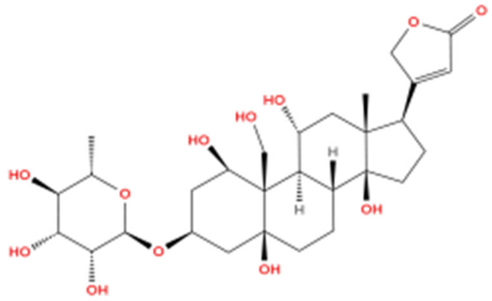

Ouabain

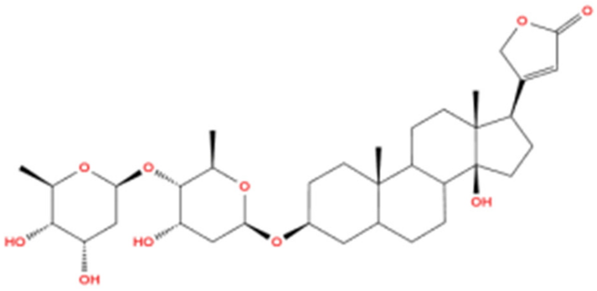

Digitoxigenin di-digitoxoside

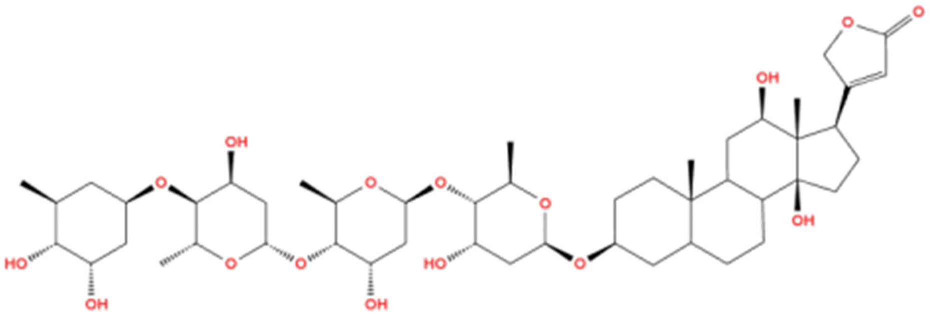

Digoxigenin tetra-digitoxose

Supporting Figure S2. Chemical structures of CTS. For all shown CTS, the C3 carbon is in the S-configuration according to R/S nomenclature and the oxygen atom bound to C3 is axial relative to ring A of the steroid core.

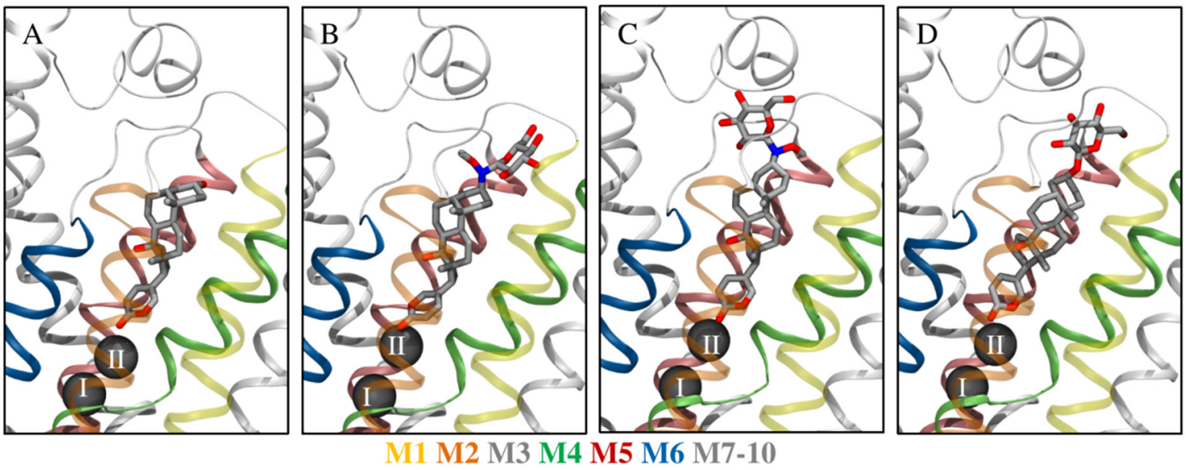

Supporting Figure S3. Starting conformations of bufalin derivatives for MD simulations. **(A)** α-bufalin, **(B)** β-bufalin-N-glucose, **(C)** α-bufalin-N-glucose, and **(D)** β-bufalin-O-glucose.

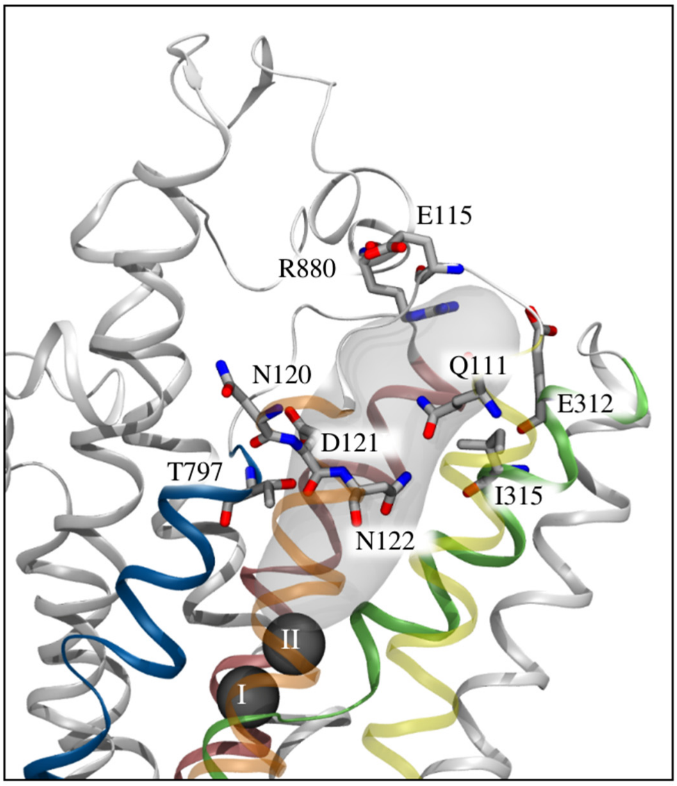

Supporting Figure S4. Overview of possible intermolecular hydrogen bonds between CTS and Na^{+},K^{+}-ATPase. The density of the lactone ring and steroid core of bufalin is shown as gray mesh.

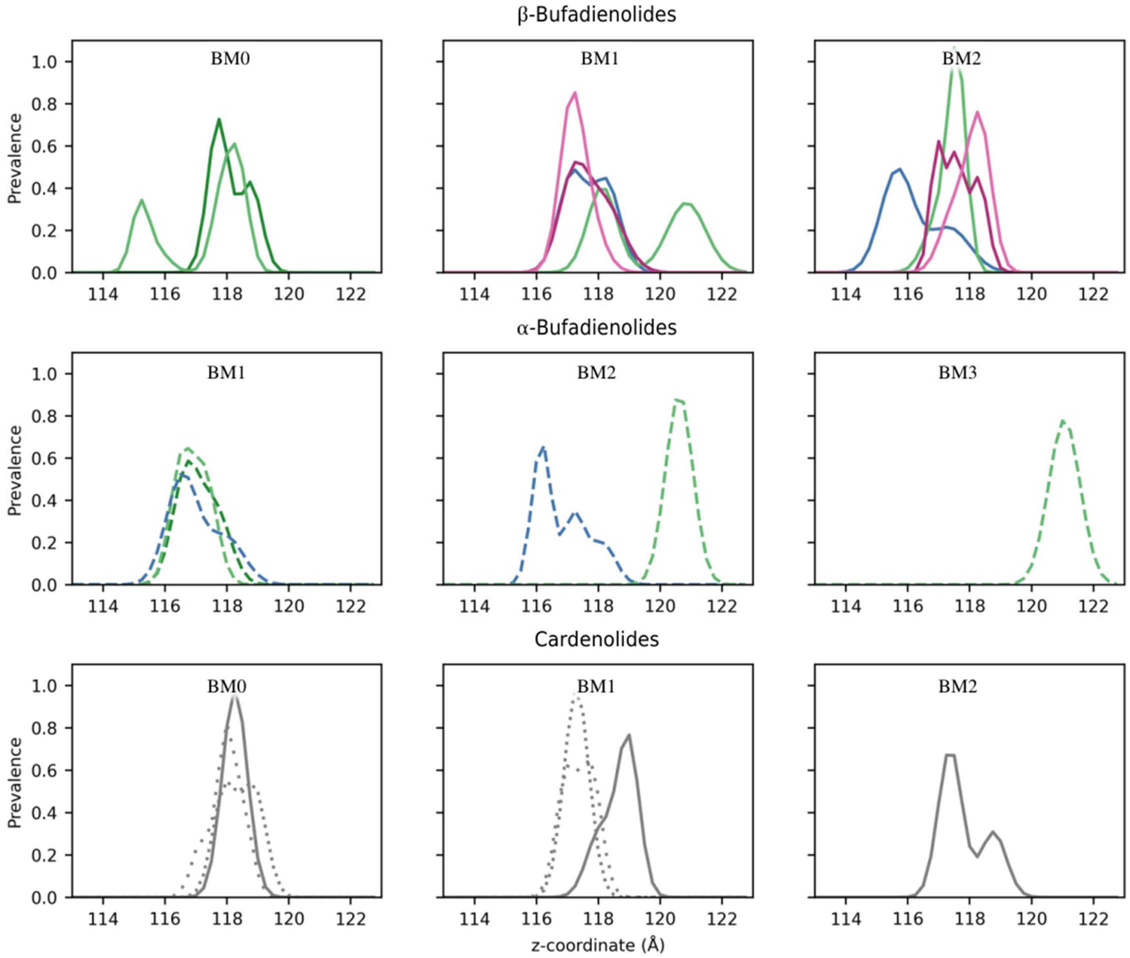

Supporting Figure S5. Ligand movement along the z-axis i.e. the longest axis of the binding site. The bufadienolides bufalin (blue), bufalin-N-glucose (green), and bufalin-O-glucose (magenta) are shown with β-isomers as solid lines and α-isomers as dashed lines. The results of simulations performed without ions in cation site I and II are shown in lighter hues. Cardenolides are all shown in gray; digoxigenin as a solid line, digoxin monodigitoxoside as a tightly dotted line, and digoxin as a loosely dotted line. Each distribution was calculated using a Gaussian Mixture model with the number of Gaussian components determined using the Bayesian information criterion. The z-coordinate represents the center-of-mass of the steroid core excl. the lactone ring. The lower the z-coord reflects position closer to the intracellular side. Before calculation, all frames were aligned by the alpha atoms of helices αM5-αM10. Only binding modes that are observed for more than 5% of the total simulation time are included.

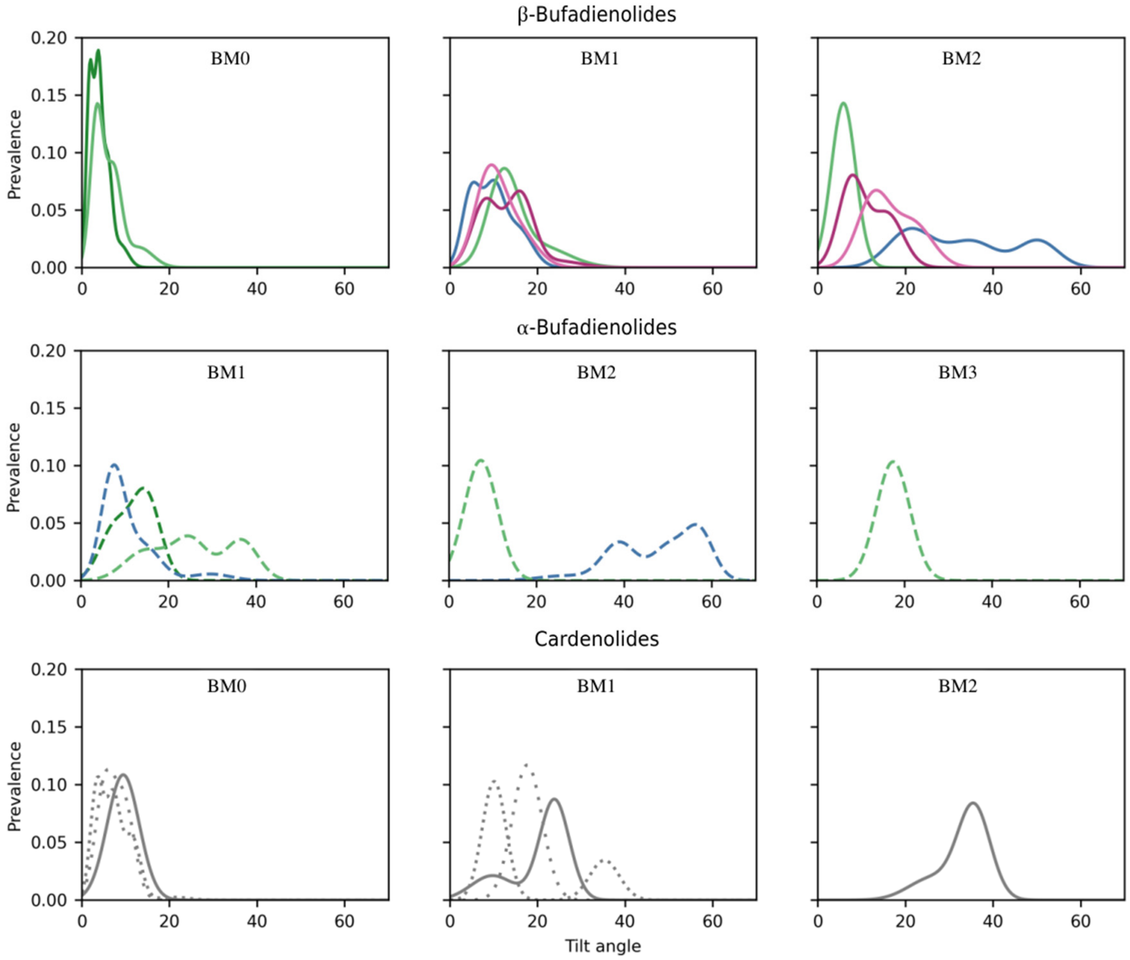

Supporting Figure S6. Distribution of tilt angles of the steroid core sampled for each ligand occupying BM0, BM1, BM2, and BM3. The bufadienolides bufalin (blue), bufalin-N-glucose (green), and bufalin-O-glucose (magenta) are shown with β-isomers as solid lines and *α*-isomers as dashed lines. Bufadienolide simulations performed without ions in cation site I and II are shown in lighter hues. Cardenolides are all shown in gray; digoxigenin as a solid line, digoxin monodigitoxoside as a tightly dotted line, and digoxin as a loosely dotted line. Each distribution was calculated using a Gaussian Mixture model with the number of Gaussian components determined using the Bayesian information criterion. Only binding modes that are observed for more than 5% of the total simulation time are included.

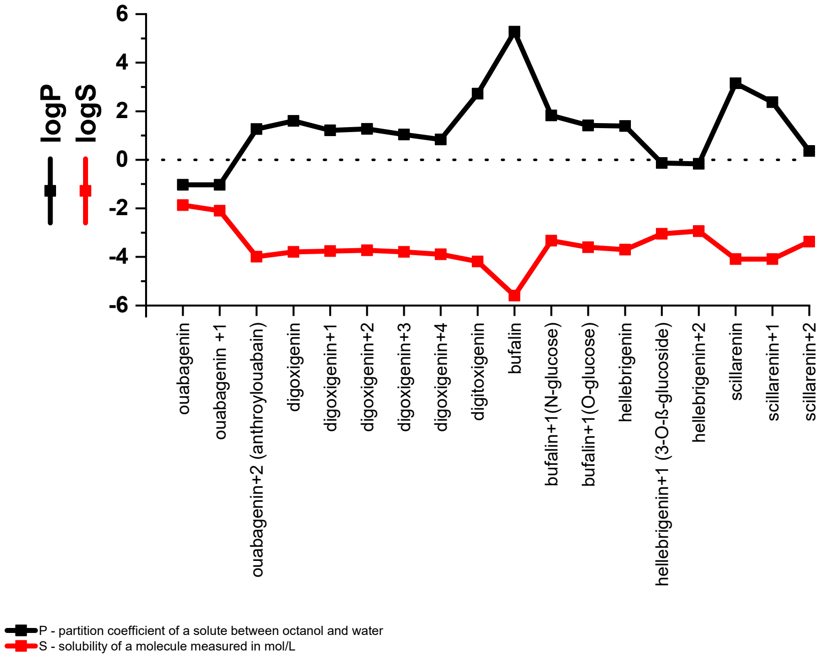

Supporting Figure S7. Estimated solubility (logS) and lipophilicity (logP) for each CTS calculated by ALOGPS 2.1.

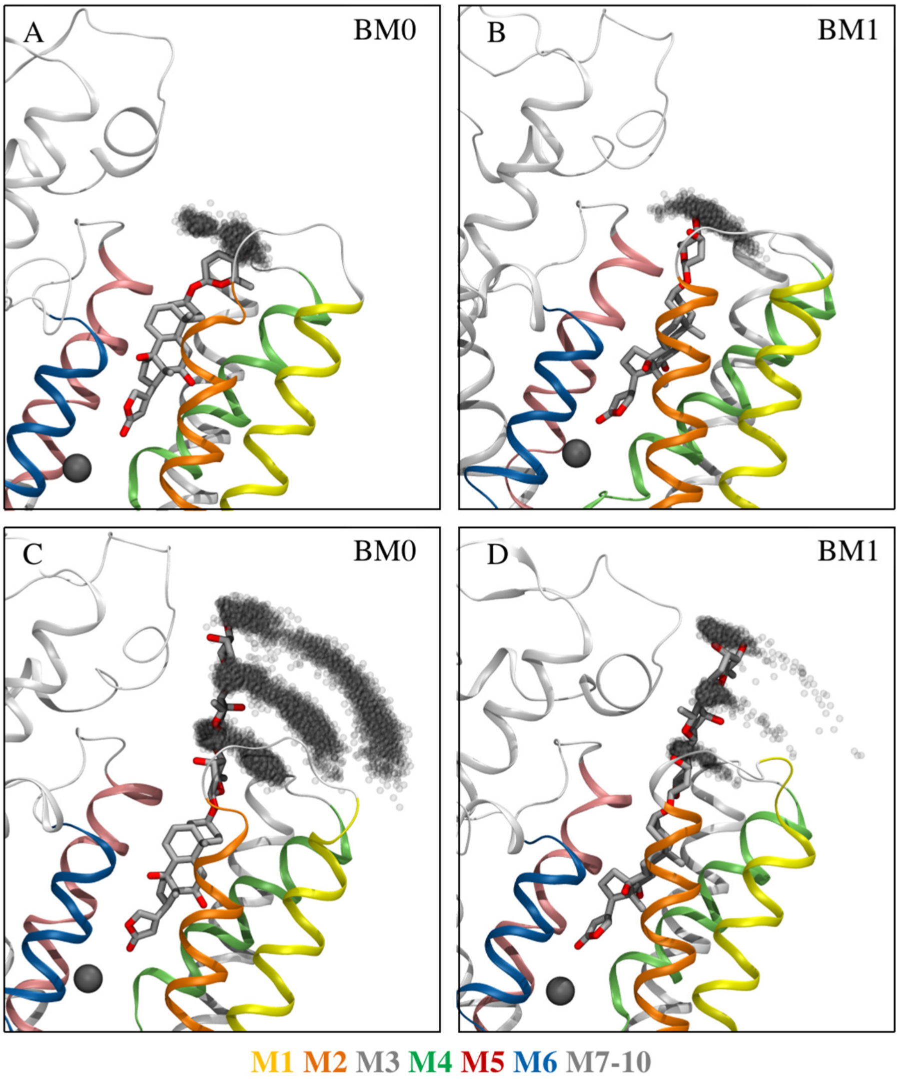

Supporting Figure S8. Dynamic range of sugar moieties in **(A-B)** digoxigenin monodigitoxoside and **(C-D)** digoxin. Each gray dot is the location of the oxygen atom of the *para*-hydroxyl group. All trajectories are aligned on M1-6, and only frames representative of a given binding mode are included in each image.

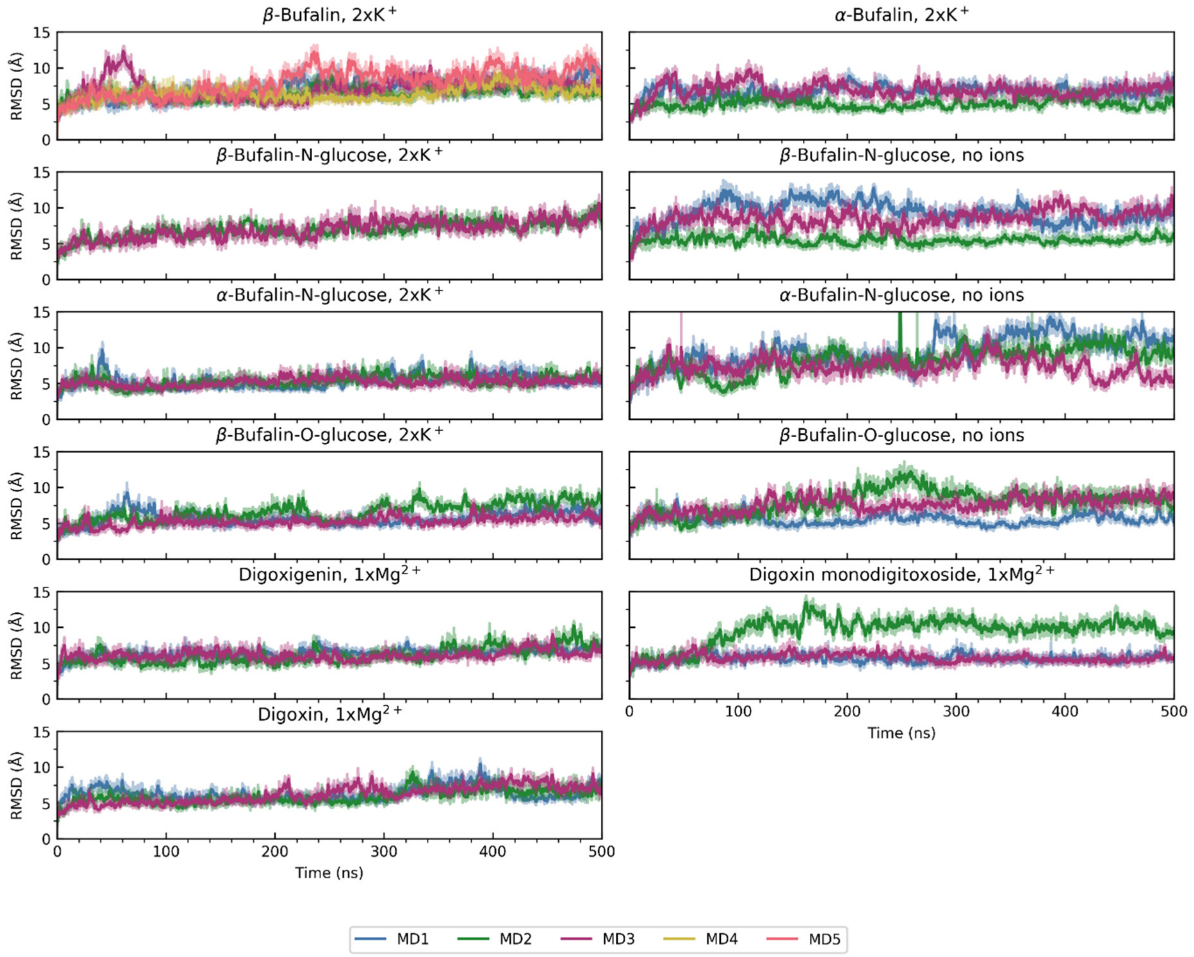

Supporting Figure S9. RMSD progression of all simulated systems. The running average is shown in solid hues, while the raw data is shown in transparent hues. The RMSD was calculated for alpha residues of chain A following alignment of residues 760 to 1016.

Supporting Data File S1. Digoxin force field parameters.

#digoxin.pdb

```
ATOM 1 C1 digoX1017 63.320 67.120 123.590 0.00 0.00
ATOM 2 C2 digoX1017 62.400 68.350 123.580 0.00 0.00
ATOM 3 C3 digoX1017 61.190 68.200 124.520 0.00 0.00
ATOM 4 O3 digoX1017 61.530 68.320 125.910 0.00 0.00
ATOM 5 C4 digoX1017 60.500 66.830 124.280 0.00 0.00
ATOM 6 C5 digoX1017 61.430 65.590 124.110 0.00 0.00
ATOM 7 C6 digoX1017 60.610 64.340 123.730 0.00 0.00
ATOM 8 C7 digoX1017 60.190 64.340 122.260 0.00 0.00
ATOM 9 C8 digoX1017 61.420 64.510 121.330 0.00 0.00
ATOM 10 C9 digoX1017 62.250 65.750 121.690 0.00 0.00
ATOM 11 C10 digoX1017 62.640 65.780 123.190 0.00 0.00
ATOM 12 C11 digoX1017 63.460 65.740 120.730 0.00 0.00
ATOM 13 C12 digoX1017 63.060 65.840 119.240 0.00 0.00
ATOM 14 O12 digoX1017 64.260 65.950 118.470 0.00 0.00
ATOM 15 C13 digoX1017 62.170 64.630 118.840 0.00 0.00
ATOM 16 C14 digoX1017 60.940 64.510 119.830 0.00 0.00
ATOM 17 O14 digoX1017 60.110 63.380 119.560 0.00 0.00
ATOM 18 C15 digoX1017 60.080 65.690 119.390 0.00 0.00
ATOM 19 C16 digoX1017 60.110 65.620 117.840 0.00 0.00
ATOM 20 C17 digoX1017 61.480 64.990 117.460 0.00 0.00
ATOM 21 C18 digoX1017 63.040 63.340 118.770 0.00 0.00
ATOM 22 C19 digoX1017 63.680 64.680 123.510 0.00 0.00
ATOM 23 C1X digoX1017 61.890 69.620 126.340 0.00 0.00
ATOM 24 C1Y digoX1017 65.170 71.890 129.930 0.00 0.00
ATOM 25 C1Z digoX1017 66.340 72.730 135.010 0.00 0.00
ATOM 26 C20 digoX1017 61.470 63.820 116.450 0.00 0.00
ATOM 27 C21 digoX1017 60.520 62.670 116.630 0.00 0.00
ATOM 28 O21 digoX1017 60.260 62.240 115.270 0.00 0.00
ATOM 29 C22 digoX1017 61.480 64.160 115.140 0.00 0.00
ATOM 30 C23 digoX1017 60.870 63.060 114.340 0.00 0.00
ATOM 31 O23 digoX1017 60.860 62.920 113.110 0.00 0.00
ATOM 32 C2X digoX1017 61.690 69.600 127.820 0.00 0.00
ATOM 33 C2Y digoX1017 65.330 73.210 130.630 0.00 0.00
ATOM 34 C2Z digoX1017 66.110 71.840 136.180 0.00 0.00
ATOM 35 C3X digoX1017 62.280 70.790 128.530 0.00 0.00
ATOM 36 O3X digoX1017 61.530 71.950 128.210 0.00 0.00
ATOM 37 C3Y digoX1017 66.190 73.160 131.870 0.00 0.00
ATOM 38 O3Y digoX1017 67.560 73.030 131.500 0.00 0.00
ATOM 39 C3Z digoX1017 65.780 72.610 137.430 0.00 0.00
ATOM 40 O3Z digoX1017 64.470 73.150 137.300 0.00 0.00
ATOM 41 C4X digoX1017 63.730 70.970 128.190 0.00 0.00
ATOM 42 O4X digoX1017 64.230 72.130 128.900 0.00 0.00
ATOM 43 C4Y digoX1017 65.760 72.040 132.780 0.00 0.00
ATOM 44 O4Y digoX1017 66.630 71.870 133.920 0.00 0.00
ATOM 45 C4Z digoX1017 66.830 73.680 137.690 0.00 0.00
ATOM 46 O4Z digoX1017 66.480 74.550 138.770 0.00 0.00
ATOM 47 C5X digoX1017 63.790 71.070 126.670 0.00 0.00
ATOM 48 O5X digoX1017 63.280 69.850 126.110 0.00 0.00
```

ATOM	49	C5Y	digoX1017	65.670	70.770	131.940	0.00	0.00
ATOM	50	O5Y	digoX1017	64.680	70.990	130.920	0.00	0.00
ATOM	51	C5Z	digoX1017	67.140	74.460	136.420	0.00	0.00
ATOM	52	O5Z	digoX1017	67.450	73.550	135.350	0.00	0.00
ATOM	53	C6X	digoX1017	65.190	71.210	126.060	0.00	0.00
ATOM	54	C6Y	digoX1017	65.260	69.510	132.710	0.00	0.00
ATOM	55	C6Z	digoX1017	68.330	75.410	136.600	0.00	0.00
ATOM	56	H11	digoX1017	63.700	67.010	124.630	0.00	0.00
ATOM	57	H12	digoX1017	64.200	67.320	122.950	0.00	0.00
ATOM	58	H21	digoX1017	62.030	68.520	122.550	0.00	0.00
ATOM	59	H22	digoX1017	62.990	69.250	123.850	0.00	0.00
ATOM	60	H3	digoX1017	60.450	69.000	124.270	0.00	0.00
ATOM	61	H41	digoX1017	59.890	66.930	123.370	0.00	0.00
ATOM	62	H42	digoX1017	59.810	66.640	125.130	0.00	0.00
ATOM	63	H5	digoX1017	61.840	65.390	125.130	0.00	0.00
ATOM	64	H61	digoX1017	59.720	64.260	124.390	0.00	0.00
ATOM	65	H62	digoX1017	61.230	63.430	123.920	0.00	0.00
ATOM	66	H71	digoX1017	59.480	65.160	122.060	0.00	0.00
ATOM	67	H72	digoX1017	59.680	63.370	122.040	0.00	0.00
ATOM	68	H8	digoX1017	62.060	63.600	121.470	0.00	0.00
ATOM	69	H9	digoX1017	61.640	66.660	121.490	0.00	0.00
ATOM	70	H111	digoX1017	64.030	64.810	120.890	0.00	0.00
ATOM	71	H112	digoX1017	64.140	66.580	120.980	0.00	0.00
ATOM	72	H1	digoX1017	62.490	66.780	119.080	0.00	0.00
ATOM	73	HO12	digoX1017	64.760	65.180	118.740	0.00	0.00
ATOM	74	HO14	digoX1017	60.580	62.570	119.840	0.00	0.00
ATOM	75	H151	digoX1017	60.470	66.640	119.750	0.00	0.00
ATOM	76	H152	digoX1017	59.050	65.620	119.780	0.00	0.00
ATOM	77	H161	digoX1017	59.980	66.620	117.400	0.00	0.00
ATOM	78	H162	digoX1017	59.260	65.020	117.490	0.00	0.00
ATOM	79	H17	digoX1017	62.090	65.810	117.040	0.00	0.00
ATOM	80	H181	digoX1017	62.430	62.440	118.630	0.00	0.00
ATOM	81	H182	digoX1017	63.770	63.410	117.940	0.00	0.00
ATOM	82	H183	digoX1017	63.610	63.180	119.710	0.00	0.00
ATOM	83	H191	digoX1017	63.360	63.690	123.120	0.00	0.00
ATOM	84	H192	digoX1017	64.660	64.920	123.050	0.00	0.00
ATOM	85	H193	digoX1017	63.830	64.580	124.600	0.00	0.00
ATOM	86	H1X	digoX1017	61.240	70.400	125.890	0.00	0.00
ATOM	87	H1Y	digoX1017	66.120	71.530	129.490	0.00	0.00
ATOM	88	H1Z	digoX1017	65.470	73.380	134.790	0.00	0.00
ATOM	89	H211	digoX1017	59.550	62.920	117.090	0.00	0.00
ATOM	90	H212	digoX1017	60.940	61.820	117.180	0.00	0.00
ATOM	91	H2	digoX1017	61.930	65.010	114.670	0.00	0.00
ATOM	92	H2X1	digoX1017	62.170	68.680	128.240	0.00	0.00
ATOM	93	H2X2	digoX1017	60.600	69.530	128.040	0.00	0.00
ATOM	94	H2Y1	digoX1017	64.310	73.560	130.940	0.00	0.00
ATOM	95	H2Y2	digoX1017	65.700	73.980	129.930	0.00	0.00
ATOM	96	H2Z1	digoX1017	67.020	71.240	136.380	0.00	0.00
ATOM	97	H2Z2	digoX1017	65.290	71.120	135.960	0.00	0.00
ATOM	98	H3X	digoX1017	62.180	70.620	129.630	0.00	0.00
ATOM	99	HO3X	digoX1017	62.090	72.740	128.350	0.00	0.00

```
ATOM    100  H3Y digoX1017      66.100  74.130 132.410  0.00  0.00
ATOM    101 HO3Y digoX1017      67.850  72.150 131.750  0.00  0.00
ATOM    102  H3Z digoX1017      65.770  71.910 138.300  0.00  0.00
ATOM    103 HO3Z digoX1017      63.900  72.660 137.940  0.00  0.00
ATOM    104  H4X digoX1017      64.310  70.080 128.500  0.00  0.00
ATOM    105  H4Y digoX1017      64.720  72.240 133.140  0.00  0.00
ATOM    106  H4Z digoX1017      67.770  73.150 137.990  0.00  0.00
ATOM    107 HO4Z digoX1017      65.670  75.030 138.520  0.00  0.00
ATOM    108  H5X digoX1017      63.180  71.930 126.310  0.00  0.00
ATOM    109  H5Y digoX1017      66.660  70.560 131.480  0.00  0.00
ATOM    110  H5Z digoX1017      66.250  75.070 136.140  0.00  0.00
ATOM    111 H6X1 digoX1017      65.100  71.300 124.960  0.00  0.00
ATOM    112 H6X2 digoX1017      65.720  72.100 126.460  0.00  0.00
ATOM    113 H6X3 digoX1017      65.800  70.300 126.280  0.00  0.00
ATOM    114 H6Y1 digoX1017      65.380  68.620 132.050  0.00  0.00
ATOM    115 H6Y2 digoX1017      65.900  69.370 133.600  0.00  0.00
ATOM    116 H6Y3 digoX1017      64.200  69.560 133.020  0.00  0.00
ATOM    117 H6Z1 digoX1017      68.540  75.970 135.660  0.00  0.00
ATOM    118 H6Z2 digoX1017      68.130  76.130 137.400  0.00  0.00
ATOM    119 H6Z3 digoX1017      69.240  74.830 136.870  0.00  0.00
END
```

#digoxin.itp excerpt

```
[ atoms ]
;          nr        type       resnr residue  atom   cgnr      charge
           mass  typeB    chargeB                  massB
; atoms w X: first sugar moiety
; atoms w Y: second/middle sugar moiety
; atoms w Z: third/terminal sugar moiety
; residue      1 digoxin rtp digoxin q      qsum
     1    CG321    1 digoxin   C1    1   -0.174   12.011  ;
     2    CG321    1 digoxin   C2    2   -0.194   12.011  ;
     3    CG311    1 digoxin   C3    3    0.090   12.011  ;
     4    OC301    1 digoxin   O3    4   -0.360   15.999  ;
     5    CG321    1 digoxin   C4    5   -0.178   12.011  ;
     6    CG311    1 digoxin   C5    6   -0.088   12.011  ;
     7    CG321    1 digoxin   C6    7   -0.180   12.011  ;
     8    CG321    1 digoxin   C7    8   -0.180   12.011  ;
     9    CG311    1 digoxin   C8    9   -0.088   12.011  ;
    10    CG311    1 digoxin   C9   10   -0.091   12.011  ;
    11    CG301    1 digoxin   C10  11   -0.005   12.011  ;
    12    CG321    1 digoxin   C11  12   -0.178   12.011  ;
    13    CG311    1 digoxin   C12  13    0.116   12.011  ;
    14    OG311    1 digoxin   O12  14   -0.649   15.999  ;
    15    CG3RC1   1 digoxin   C13  15    0.040   12.011  ;
    16    CG3RC1   1 digoxin   C14  16    0.238   12.011  ;
    17    OG311    1 digoxin   O14  17   -0.652   15.999  ;
    18    CG3C52   1 digoxin   C15  18   -0.200   12.011  ;
    19    CG3C52   1 digoxin   C16  19   -0.176   12.011  ;
    20    CG3C51   1 digoxin   C17  20   -0.095   12.011  ;
    21    CG331    1 digoxin   C18  21   -0.287   12.011  ;
```

22	CG331	1	digoxin	C19	22	-0.273	12.011	;
23	CC3162	1	digoxin	C1X	23	0.290	12.011	;
24	CC3162	1	digoxin	C1Y	24	0.290	12.011	;
25	CC3162	1	digoxin	C1Z	25	0.290	12.011	;
26	CG2R57	1	digoxin	C20	26	-0.013	12.011	;
27	CG3C52	1	digoxin	C21	27	0.230	12.011	;
28	OG3C51	1	digoxin	O21	28	-0.307	15.999	;
29	CG2R51	1	digoxin	C22	29	-0.188	12.011	;
30	CG2R53	1	digoxin	C23	30	0.290	12.011	;
31	OG2D1	1	digoxin	O23	31	-0.430	15.999	;
32	CC3261	1	digoxin	C2X	32	-0.180	12.011	;
33	CC3261	1	digoxin	C2Y	33	-0.180	12.011	;
34	CC3261	1	digoxin	C2Z	34	-0.180	12.011	;
35	CC3161	1	digoxin	C3X	35	0.140	12.011	;
36	OC311	1	digoxin	O3X	36	-0.650	15.999	;
37	CC3161	1	digoxin	C3Y	37	0.140	12.011	;
38	OC311	1	digoxin	O3Y	38	-0.650	15.999	;
39	CC3161	1	digoxin	C3Z	39	0.140	12.011	;
40	OC311	1	digoxin	O3Z	40	-0.650	15.999	;
41	CC3161	1	digoxin	C4X	41	0.090	12.011	;
42	OC301	1	digoxin	O4X	42	-0.360	15.999	;
43	CC3161	1	digoxin	C4Y	43	0.090	12.011	;
44	OC301	1	digoxin	O4Y	44	-0.360	15.999	;
45	CC3161	1	digoxin	C4Z	45	0.140	12.011	;
46	OC311	1	digoxin	O4Z	46	-0.650	15.999	;
47	CC3163	1	digoxin	C5X	47	0.110	12.011	;
48	OC3C61	1	digoxin	O5X	48	-0.400	15.999	;
49	CC3163	1	digoxin	C5Y	49	0.110	12.011	;
50	OC3C61	1	digoxin	O5Y	50	-0.400	15.999	;
51	CC3163	1	digoxin	C5Z	51	0.110	12.011	;
52	OC3C61	1	digoxin	O5Z	52	-0.400	15.999	;
53	CC331	1	digoxin	C6X	53	-0.270	12.011	;
54	CC331	1	digoxin	C6Y	54	-0.270	12.011	;
55	CC331	1	digoxin	C6Z	55	-0.270	12.011	;
56	HGA2	1	digoxin	H11	56	0.090	1.008	;
57	HGA2	1	digoxin	H12	57	0.090	1.008	;
58	HGA2	1	digoxin	H21	58	0.090	1.008	;
59	HGA2	1	digoxin	H22	59	0.090	1.008	;
60	HGA1	1	digoxin	H3	60	0.090	1.008	;
61	HGA2	1	digoxin	H41	61	0.090	1.008	;
62	HGA2	1	digoxin	H42	62	0.090	1.008	;
63	HGA1	1	digoxin	H5	63	0.090	1.008	;
64	HGA2	1	digoxin	H61	64	0.090	1.008	;
65	HGA2	1	digoxin	H62	65	0.090	1.008	;
66	HGA2	1	digoxin	H71	66	0.090	1.008	;
67	HGA2	1	digoxin	H72	67	0.090	1.008	;
68	HGA1	1	digoxin	H8	68	0.090	1.008	;
69	HGA1	1	digoxin	H9	69	0.090	1.008	;
70	HGA2	1	digoxin	H111	70	0.090	1.008	;
71	HGA2	1	digoxin	H112	71	0.090	1.008	;
72	HGA1	1	digoxin	H1	72	0.090	1.008	;

```
73   HGP1   1 digoxin  HO12   73   0.419   1.008  ;
74   HGP1   1 digoxin  HO14   74   0.420   1.008  ;
75   HGA2   1 digoxin  H151   75   0.090   1.008  ;
76   HGA2   1 digoxin  H152   76   0.090   1.008  ;
77   HGA2   1 digoxin  H161   77   0.090   1.008  ;
78   HGA2   1 digoxin  H162   78   0.090   1.008  ;
79   HGA1   1 digoxin  H17    79   0.090   1.008  ;
80   HGA3   1 digoxin  H181   80   0.090   1.008  ;
81   HGA3   1 digoxin  H182   81   0.090   1.008  ;
82   HGA3   1 digoxin  H183   82   0.090   1.008  ;
83   HGA3   1 digoxin  H191   83   0.090   1.008  ;
84   HGA3   1 digoxin  H192   84   0.090   1.008  ;
85   HGA3   1 digoxin  H193   85   0.090   1.008  ;
86   HCA1   1 digoxin  H1X    86   0.090   1.008  ;
87   HCA1   1 digoxin  H1Y    87   0.090   1.008  ;
88   HCA1   1 digoxin  H1Z    88   0.090   1.008  ;
89   HGA2   1 digoxin  H211   89   0.090   1.008  ;
90   HGA2   1 digoxin  H212   90   0.090   1.008  ;
91   HGR51  1 digoxin  H2     91   0.263   1.008  ;
92   HCA2   1 digoxin  H2X1   92   0.090   1.008  ;
93   HCA2   1 digoxin  H2X2   93   0.090   1.008  ;
94   HCA2   1 digoxin  H2Y1   94   0.090   1.008  ;
95   HCA2   1 digoxin  H2Y2   95   0.090   1.008  ;
96   HCA2   1 digoxin  H2Z1   96   0.090   1.008  ;
97   HCA2   1 digoxin  H2Z2   97   0.090   1.008  ;
98   HCA1   1 digoxin  H3X    98   0.090   1.008  ;
99   HCP1   1 digoxin  HO3X   99   0.420   1.008  ;
100  HCA1   1 digoxin  H3Y    100  0.090   1.008  ;
101  HCP1   1 digoxin  HO3Y   101  0.420   1.008  ;
102  HCA1   1 digoxin  H3Z    102  0.090   1.008  ;
103  HCP1   1 digoxin  HO3Z   103  0.420   1.008  ;
104  HCA1   1 digoxin  H4X    104  0.090   1.008  ;
105  HCA1   1 digoxin  H4Y    105  0.090   1.008  ;
106  HCA1   1 digoxin  H4Z    106  0.090   1.008  ;
107  HCP1   1 digoxin  HO4Z   107  0.420   1.008  ;
108  HCA1   1 digoxin  H5X    108  0.090   1.008  ;
109  HCA1   1 digoxin  H5Y    109  0.090   1.008  ;
110  HCA1   1 digoxin  H5Z    110  0.090   1.008  ;
111  HCA3   1 digoxin  H6X1   111  0.090   1.008  ;
112  HCA3   1 digoxin  H6X2   112  0.090   1.008  ;
113  HCA3   1 digoxin  H6X3   113  0.090   1.008  ;
114  HCA3   1 digoxin  H6Y1   114  0.090   1.008  ;
115  HCA3   1 digoxin  H6Y2   115  0.090   1.008  ;
116  HCA3   1 digoxin  H6Y3   116  0.090   1.008  ;
117  HCA3   1 digoxin  H6Z1   117  0.090   1.008  ;
118  HCA3   1 digoxin  H6Z2   118  0.090   1.008  ;
119  HCA3   1 digoxin  H6Z3   119  0.090   1.008  ;
```

#digoxin.prm

```
[ bondtypes ]
;    i    j  func     b0      kb
```

```
  CG2R57   CG3C51    1   0.15100000    292880.00
  CG2R57   CG3C52    1   0.15100000    292880.00
  CG3RC1   OG311    1   0.14200000    358150.40
  CC3261   CC3162    1   0.14800000    186188.00
   CG311   OC301    1   0.14150000    301248.00

[ angletypes ]
;    i     j     k  func     theta0     ktheta       ub0       kub
  CG2R53  CG2R51  CG2R57    5  116.500000  836.800000  0.00000000      0.00
  CG2R51  CG2R57  CG3C51    5  109.000000  962.320000  0.00000000      0.00
  CG2R51  CG2R57  CG3C52    5  109.000000  962.320000  0.00000000      0.00
  CG3C51  CG2R57  CG3C52    5  105.800000  753.120000  0.00000000      0.00
   CG311   CG311   OC311    5  109.700000  962.320000  0.00000000      0.00
   CG311   CG311  OG3C61    5  111.500000  376.560000  0.00000000      0.00
   CG321   CG311   OC311    5  109.700000  962.320000  0.00000000      0.00
   CG321   CG311   OC301    5  109.000000  376.560000  0.00000000      0.00
   CG321   CG311  OG3C61    5  111.500000  376.560000  0.00000000      0.00
   CG331   CG311  OG3C61    5  111.500000  376.560000  0.00000000      0.00
  OG3C61   CG311    HGA1    5  109.500000  376.560000  0.00000000      0.00
  CG2R57  CG3C51  CG3C52    5  106.000000  435.136000  0.00000000      0.00
  CG2R57  CG3C51  CG3RC1    5  106.000000  435.136000  0.00000000      0.00
  CG2R57  CG3C51    HGA1    5  112.600000  435.136000  0.00000000      0.00
  CG2R57  CG3C52  OG3C51    5  102.400000  627.600000  0.00000000      0.00
  CG2R57  CG3C52    HGA2    5  112.600000  435.136000  0.00000000      0.00
   CG311  CG3RC1   OG311    5  113.500000  488.272800  0.25610000    9338.69
  CG3C52  CG3RC1   OG311    5  110.000000  633.457600  0.00000000      0.00
  CG3RC1  CG3RC1   OG311    5  111.000000  446.432800  0.25610000    6694.40
  CC3162   OC301   CG311    5  109.200000  418.400000  0.00000000      0.00
  CG3RC1   OG311    HGP1    5  109.000000  418.400000  0.00000000      0.00
   CG311  OG3C61   CG311    5  109.700000  794.960000  0.00000000      0.00
  CC3261  CC3162    HCA1    5  110.100000  288.696000  0.21790000   18853.10
  CC3261  CC3162  OC3C61    5  106.000000  376.560000  0.00000000      0.00
   OC301   CG311    HGA1    5  109.500000  502.080000  0.00000000      0.00
  CC3261  CC3162   OC311    5  107.000000  633.457600  0.00000000      0.00
   OC301  CC3162  CC3261    5  105.000000  376.560000  0.00000000      0.00
  CC3162  CC3261  CC3161    5  111.000000  446.432800  0.25610000    6694.40
  CC3162  CC3261    HCA2    5  110.100000  288.696000  0.21790000   18853.10

[ dihedraltypes ]
;    i     j     k     l  func      phi0      kphi  mult
  CG2R57  CG2R51  CG2R53   OG2D1    9  180.000000    5.020800    2
  CG2R57  CG2R51  CG2R53  OG3C51    9  180.000000    1.715440    1
  CG2R53  CG2R51  CG2R57  CG3C51    9  180.000000   27.614400    2
  CG2R53  CG2R51  CG2R57  CG3C52    9  180.000000   27.614400    2
   HGR51  CG2R51  CG2R57  CG3C51    9  180.000000   12.133600    2
   HGR51  CG2R51  CG2R57  CG3C52    9  180.000000   12.133600    2
  CG2R51  CG2R57  CG3C51  CG3C52    9  180.000000    0.209200    3
  CG2R51  CG2R57  CG3C51  CG3RC1    9  180.000000    0.209200    3
  CG2R51  CG2R57  CG3C51    HGA1    9    0.000000    0.000000    3
  CG3C52  CG2R57  CG3C51  CG3C52    9    0.000000    9.916080    2
  CG3C52  CG2R57  CG3C51  CG3RC1    9    0.000000    9.916080    2
```

CG3C52	CG2R57	CG3C51	HGA1	9	180.000000	0.000000	3
CG2R51	CG2R57	CG3C52	OG3C51	9	180.000000	1.171520	1
CG2R51	CG2R57	CG3C52	OG3C51	9	180.000000	4.100320	2
CG2R51	CG2R57	CG3C52	OG3C51	9	180.000000	7.322000	3
CG2R51	CG2R57	CG3C52	HGA2	9	0.000000	0.000000	3
CG3C51	CG2R57	CG3C52	OG3C51	9	180.000000	1.171520	1
CG3C51	CG2R57	CG3C52	OG3C51	9	180.000000	4.100320	2
CG3C51	CG2R57	CG3C52	OG3C51	9	180.000000	7.322000	3
CG3C51	CG2R57	CG3C52	HGA2	9	180.000000	0.000000	3
CG311	CG311	CG311	CG331	9	180.000000	2.092000	4
CG311	CG311	CG311	OG3C61	9	180.000000	0.794960	1
CG311	CG311	CG311	OG3C61	9	180.000000	4.184000	2
CG311	CG311	CG311	OG3C61	9	0.000000	2.510400	3
CG311	CG311	CG311	OG3C61	9	180.000000	0.334720	4
CG321	CG311	CG311	OG301	9	180.000000	0.836800	3
CG321	CG311	CG311	OC301	9	180.000000	0.836800	3
CG321	CG311	CG311	OG311	9	0.000000	0.585760	3
CG331	CG311	CG311	OG311	9	0.000000	0.585760	3
OG301	CG311	CG311	OG311	9	0.000000	0.836800	3
OG301	CG311	CG311	OG3C61	9	0.000000	0.836800	3
OC301	CG311	CG311	OG3C61	9	0.000000	0.836800	3
OG301	CG311	CG311	HGA1	9	0.000000	0.815880	3
OC301	CG311	CG311	HGA1	9	0.000000	0.815880	3
OG311	CG311	CG311	OG311	9	0.000000	0.836800	3
OG311	CG311	CG311	OG3C61	9	0.000000	0.836800	3
OG301	CG311	CG321	CG311	9	180.000000	0.669440	1
OG301	CG311	CG321	CG311	9	0.000000	1.631760	2
OC301	CG311	CG321	CG311	9	0.000000	0.836800	3
OC301	CG311	CG321	CG321	9	0.000000	0.836800	3
OG301	CG311	CG321	HGA2	9	0.000000	0.815880	3
OC301	CG311	CG321	HGA2	9	0.000000	0.815880	3
CG311	CG311	CG3RC1	OG311	9	0.000000	0.209200	3
CG321	CG311	CG3RC1	OG311	9	0.000000	0.209200	3
HGA1	CG311	CG3RC1	OG311	9	0.000000	0.209200	3
CG311	CG311	OG301	CG311	9	0.000000	1.673600	1
CG311	CG311	OG301	CG311	9	0.000000	2.050160	3
CG321	CG311	OC301	CC3162	9	180.000000	0.543920	1
CG321	CG311	OC301	CC3162	9	180.000000	1.046000	2
CG321	CG311	OC301	CC3162	9	180.000000	0.251040	3
HGA1	CG311	OG301	CG311	9	0.000000	1.188256	3
HGA1	CG311	OC301	CC3162	9	0.000000	1.188256	3
CG2R57	CG3C51	CG3C52	CG3C52	9	180.000000	15.480800	3
CG2R57	CG3C51	CG3C52	CG3C52	9	0.000000	9.414000	4
CG2R57	CG3C51	CG3C52	CG3C52	9	180.000000	0.962320	6
CG2R57	CG3C51	CG3C52	HGA2	9	0.000000	0.585760	3
CG2R57	CG3C51	CG3RC1	CG311	9	180.000000	15.480800	3
CG2R57	CG3C51	CG3RC1	CG311	9	0.000000	9.414000	4
CG2R57	CG3C51	CG3RC1	CG311	9	180.000000	0.962320	6
CG2R57	CG3C51	CG3RC1	CG331	9	180.000000	15.480800	3
CG2R57	CG3C51	CG3RC1	CG331	9	0.000000	9.414000	4
CG2R57	CG3C51	CG3RC1	CG331	9	180.000000	0.962320	6

```
CG2R57 CG3C51 CG3RC1 CG3RC1 9 0.000000 0.627600 3
CG3C52 CG3C52 CG3RC1 OG311 9 180.000000 2.092000 1
CG3C52 CG3C52 CG3RC1 OG311 9 0.000000 2.928800 2
CG3C52 CG3C52 CG3RC1 OG311 9 0.000000 1.673600 3
CG3C52 CG3C52 CG3RC1 OG311 9 0.000000 1.673600 5
HGA2 CG3C52 CG3RC1 OG311 9 180.000000 0.815880 3
CG2R57 CG3C52 OG3C51 CG2R53 9 180.000000 1.297040 1
CG2R57 CG3C52 OG3C51 CG2R53 9 0.000000 1.171520 6
CG311 CG3RC1 CG3RC1 OG311 9 0.000000 16.736000 3
CG331 CG3RC1 CG3RC1 OG311 9 0.000000 0.209200 3
CG3C51 CG3RC1 CG3RC1 OG311 9 0.000000 5.020800 3
CG311 CG3RC1 OG311 HGP1 9 0.000000 1.213360 1
CG311 CG3RC1 OG311 HGP1 9 0.000000 2.594080 2
CG311 CG3RC1 OG311 HGP1 9 0.000000 0.209200 3
CG3C52 CG3RC1 OG311 HGP1 9 0.000000 1.213360 1
CG3C52 CG3RC1 OG311 HGP1 9 0.000000 2.594080 2
CG3C52 CG3RC1 OG311 HGP1 9 0.000000 0.209200 3
CG3RC1 CG3RC1 OG311 HGP1 9 0.000000 6.276000 1
CG3RC1 CG3RC1 OG311 HGP1 9 180.000000 1.255200 2
CG3RC1 CG3RC1 OG311 HGP1 9 0.000000 1.338880 3
CG311 OC301 CC3162 OC3C61 9 180.000000 0.209200 1
CG311 OC301 CC3162 OC3C61 9 0.000000 3.807440 2
CG311 OC301 CC3162 OC3C61 9 180.000000 5.313680 3
CC3161 CC3261 CC3162 OC301 9 0.000000 0.836800 3
CC3261 CC3162 OC301 CG311 9 180.000000 1.715440 1
CC3261 CC3162 OC301 CG311 9 180.000000 2.761440 2
CC3261 CC3162 OC301 CG311 9 0.000000 6.694400 3
CG311 OC301 CC3162 HCA1 9 0.000000 1.188256 3
CC3161 CC3261 CC3162 OC3C61 9 180.000000 1.297040 3
CC3161 CC3162 OC3C61 CC3163 9 0.000000 0.836800 3
HCA1 CC3261 CC3162 OC3C61 9 0.000000 0.836800 3
CC3261 CC3162 OC3C61 CC3163 9 0.000000 0.836800 3
HCA2 CC3261 CC3162 OC3C61 9 0.000000 0.836800 3
HCA2 CC3261 CC3162 OC301 9 0.000000 0.836800 3
HCA1 CC3162 CC3261 CC3161 9 0.000000 0.836800 3
HCA1 CC3162 CC3261 HCA2 9 0.000000 0.836800 3
CC3261 CC3162 OC301 CC3161 9 180.000000 1.715440 1
CC3261 CC3162 OC301 CC3161 9 180.000000 2.761440 2
CC3261 CC3162 OC301 CC3161 9 0.000000 6.694400 3
CC3161 CC3161 CC3261 CC3162 9 180.000000 0.794960 3
HCA1 CC3161 CC3261 CC3162 9 0.000000 0.836800 3
CC3162 CC3261 CC3161 OC311 9 0.000000 0.836800 3
CC3261 CC3161 CC3161 OC301 9 0.000000 0.836800 3
CC3261 CC3161 CC3161 OC311 9 0.000000 0.836800 3

[ dihedraltypes ]
; 'improper' dihedrals
; i j k l func phi0 kphi
```

Supporting Data File S2. Bufalin-N-glucose force field parameters.

#bufalin-N-glucose.pdb

```
ATOM      1  C18 bufaX2005      62.520  63.310 118.420  0.00  0.00
ATOM      2  C13 bufaX2005      61.600  64.550 118.520  0.00  0.00
ATOM      3  C12 bufaX2005      62.380  65.810 118.930  0.00  0.00
ATOM      4  C11 bufaX2005      62.890  65.720 120.360  0.00  0.00
ATOM      5  C9  bufaX2005      61.740  65.590 121.370  0.00  0.00
ATOM      6  C10 bufaX2005      62.210  65.470 122.840  0.00  0.00
ATOM      7  C19 bufaX2005      63.150  64.240 122.960  0.00  0.00
ATOM      8  C1  bufaX2005      63.050  66.690 123.310  0.00  0.00
ATOM      9  C2  bufaX2005      62.280  68.000 123.560  0.00  0.00
ATOM     10  C3  bufaX2005      61.160  67.880 124.620  0.00  0.00
ATOM     11  N11 bufaX2005      61.590  67.720 126.080  0.00  0.00
ATOM     12  C4  bufaX2005      60.260  66.700 124.130  0.00  0.00
ATOM     13  C5  bufaX2005      61.020  65.370 123.810  0.00  0.00
ATOM     14  C6  bufaX2005      60.040  64.250 123.390  0.00  0.00
ATOM     15  C7  bufaX2005      59.620  64.320 121.920  0.00  0.00
ATOM     16  C8  bufaX2005      60.870  64.360 121.000  0.00  0.00
ATOM     17  C14 bufaX2005      60.390  64.360 119.510  0.00  0.00
ATOM     18  O14 bufaX2005      59.680  63.160 119.200  0.00  0.00
ATOM     19  C15 bufaX2005      59.470  65.520 119.160  0.00  0.00
ATOM     20  C16 bufaX2005      59.710  65.810 117.660  0.00  0.00
ATOM     21  C17 bufaX2005      60.880  64.890 117.180  0.00  0.00
ATOM     22  C20 bufaX2005      60.510  63.750 116.260  0.00  0.00
ATOM     23  C22 bufaX2005      61.450  63.240 115.380  0.00  0.00
ATOM     24  C23 bufaX2005      61.200  62.190 114.510  0.00  0.00
ATOM     25  C24 bufaX2005      59.920  61.590 114.510  0.00  0.00
ATOM     26  O24 bufaX2005      59.500  60.830 113.640  0.00  0.00
ATOM     27  O21 bufaX2005      58.960  62.360 115.190  0.00  0.00
ATOM     28  C21 bufaX2005      59.270  63.170 116.270  0.00  0.00
ATOM     29 H181 bufaX2005      62.830  62.970 119.430  0.00  0.00
ATOM     30 H182 bufaX2005      62.020  62.460 117.920  0.00  0.00
ATOM     31 H183 bufaX2005      63.450  63.550 117.860  0.00  0.00
ATOM     32 H121 bufaX2005      63.260  65.910 118.260  0.00  0.00
ATOM     33 H122 bufaX2005      61.770  66.730 118.860  0.00  0.00
ATOM     34 H111 bufaX2005      63.490  66.630 120.570  0.00  0.00
ATOM     35 H112 bufaX2005      63.560  64.850 120.400  0.00  0.00
ATOM     36  H9  bufaX2005      61.110  66.500 121.300  0.00  0.00
ATOM     37 H191 bufaX2005      63.670  64.240 123.930  0.00  0.00
ATOM     38 H192 bufaX2005      62.590  63.290 122.880  0.00  0.00
ATOM     39 H193 bufaX2005      63.950  64.230 122.210  0.00  0.00
ATOM     40  H11 bufaX2005      63.480  66.410 124.290  0.00  0.00
ATOM     41  H12 bufaX2005      63.880  66.860 122.600  0.00  0.00
ATOM     42  H21 bufaX2005      61.800  68.280 122.600  0.00  0.00
ATOM     43  H22 bufaX2005      62.940  68.830 123.810  0.00  0.00
ATOM     44  H3  bufaX2005      60.560  68.810 124.610  0.00  0.00
ATOM     45  H41 bufaX2005      59.750  67.020 123.200  0.00  0.00
ATOM     46  H42 bufaX2005      59.470  66.500 124.870  0.00  0.00
ATOM     47  H5  bufaX2005      61.420  65.120 124.810  0.00  0.00
ATOM     48  H61 bufaX2005      60.500  63.250 123.570  0.00  0.00
```

```
ATOM     49  H62 bufaX2005      59.130  64.300 124.030  0.00  0.00
ATOM     50  H71 bufaX2005      59.000  65.230 121.770  0.00  0.00
ATOM     51  H72 bufaX2005      58.990  63.430 121.680  0.00  0.00
ATOM     52  H8  bufaX2005     61.440  63.420 121.140  0.00  0.00
ATOM     53 HO14 bufaX2005      60.310  62.450 119.030  0.00  0.00
ATOM     54 H151 bufaX2005      59.700  66.400 119.770  0.00  0.00
ATOM     55 H152 bufaX2005      58.420  65.280 119.370  0.00  0.00
ATOM     56 H161 bufaX2005      59.960  66.870 117.530  0.00  0.00
ATOM     57 H162 bufaX2005      58.790  65.650 117.090  0.00  0.00
ATOM     58  H17 bufaX2005      61.570  65.530 116.610  0.00  0.00
ATOM     59  H2  bufaX2005     62.440  63.710 115.380  0.00  0.00
ATOM     60  H23 bufaX2005      61.940  61.870 113.780  0.00  0.00
ATOM     61  H1  bufaX2005     58.570  63.380 117.080  0.00  0.00
ATOM     62  O62 bufaX2005      60.420  67.520 126.860  0.00  0.00
ATOM     63  C63 bufaX2005      60.490  66.370 127.690  0.00  0.00
ATOM     64  H64 bufaX2005      59.560  66.270 128.290  0.00  0.00
ATOM     65  H65 bufaX2005      60.580  65.450 127.070  0.00  0.00
ATOM     66  H66 bufaX2005      61.350  66.430 128.390  0.00  0.00
ATOM     67  H67 bufaX2005      62.530  68.380 127.860  0.00  0.00
ATOM     68  C68 bufaX2005      62.360  70.810 128.000  0.00  0.00
ATOM     69  O69 bufaX2005      61.870  70.070 126.860  0.00  0.00
ATOM     70  C70 bufaX2005      62.470  68.760 126.810  0.00  0.00
ATOM     71  C71 bufaX2005      63.920  68.980 126.400  0.00  0.00
ATOM     72  C72 bufaX2005      64.690  69.980 127.270  0.00  0.00
ATOM     73  C73 bufaX2005      63.860  71.100 127.870  0.00  0.00
ATOM     74  H74 bufaX2005      62.200  70.190 128.910  0.00  0.00
ATOM     75  C75 bufaX2005      61.530  72.070 128.180  0.00  0.00
ATOM     76  O76 bufaX2005      64.640  67.760 126.390  0.00  0.00
ATOM     77  H77 bufaX2005      63.940  69.420 125.380  0.00  0.00
ATOM     78  H78 bufaX2005      65.110  69.380 128.110  0.00  0.00
ATOM     79  H79 bufaX2005      63.940  71.980 127.190  0.00  0.00
ATOM     80  O80 bufaX2005      64.390  71.450 129.140  0.00  0.00
ATOM     81  H81 bufaX2005      61.790  72.820 127.400  0.00  0.00
ATOM     82  O82 bufaX2005      61.670  72.680 129.470  0.00  0.00
ATOM     83  H83 bufaX2005      60.460  71.800 128.060  0.00  0.00
ATOM     84  H84 bufaX2005      62.540  73.150 129.560  0.00  0.00
ATOM     85  H85 bufaX2005      64.630  67.370 127.300  0.00  0.00
ATOM     86  H86 bufaX2005      64.280  70.670 129.720  0.00  0.00
ATOM     87  O87 bufaX2005      65.850  70.500 126.610  0.00  0.00
ATOM     88  H88 bufaX2005      66.510  70.710 127.280  0.00  0.00
END
```

#**bufalin-N-glucose.itp excerpt**

```
[ atoms ]

;   nr   type   resnr   residue   atom   cgnr
    charge   mass   typeB   chargeB   massB
;   residue   1   bufalin-N-glucose   rtp   bufalin-N-
glucose   q   qsum
    1   CTL3   1   bufalin-N-glucose   C18
    1   -0.268   12.011
```

2	CRL1	1	bufalin-N-glucose	C13
2	0.001	12.011		
3	CRL2	1	bufalin-N-glucose	C12
3	-0.183	12.011		
4	CRL2	1	bufalin-N-glucose	C11
4	-0.182	12.011		
5	CRL1	1	bufalin-N-glucose	C9
5	-0.09	12.011		
6	CRL1	1	bufalin-N-glucose	C10
6	-0.001	12.011		
7	CTL3	1	bufalin-N-glucose	C19
7	-0.274	12.011		
8	CRL2	1	bufalin-N-glucose	C1
8	-0.18	12.011		
9	CRL2	1	bufalin-N-glucose	C2
9	-0.179	12.011		
10	CRL1	1	bufalin-N-glucose	C3
10	0.135	12.011		
11	NG301	1	bufalin-N-glucose	N11
11	-0.391	14.007		
12	CRL2	1	bufalin-N-glucose	C4
12	-0.177	12.011		
13	CRL1	1	bufalin-N-glucose	C5
13	-0.09	12.011		
14	CRL2	1	bufalin-N-glucose	C6
14	-0.181	12.011		
15	CRL2	1	bufalin-N-glucose	C7
15	-0.181	12.011		
16	CRL1	1	bufalin-N-glucose	C8
16	-0.09	12.011		
17	CRL1	1	bufalin-N-glucose	C14
17	0.238	12.011		
18	OHL	1	bufalin-N-glucose	O14
18	-0.651	15.999		
19	CRL2	1	bufalin-N-glucose	C15
19	-0.181	12.011		
20	CRL2	1	bufalin-N-glucose	C16
20	-0.177	12.011		
21	CRL1	1	bufalin-N-glucose	C17
21	-0.039	12.011		
22	CG2R62	1	bufalin-N-glucose	C20
22	0.032	12.011		
23	CG2R62	1	bufalin-N-glucose	C22
23	-0.272	12.011		
24	CG2R62	1	bufalin-N-glucose	C23
24	-0.347	12.011		
25	CG2R63	1	bufalin-N-glucose	C24
25	0.49	12.011		
26	OG2D4	1	bufalin-N-glucose	O24
26	-0.46	15.999		

27	OG3R60	1	bufalin-N-glucose	O21
27	-0.362	15.999		
28	CG2R62	1	bufalin-N-glucose	C21
28	0.119	12.011		
29	HAL3	1	bufalin-N-glucose	H181
29	0.09	1.008		
30	HAL3	1	bufalin-N-glucose	H182
30	0.09	1.008		
31	HAL3	1	bufalin-N-glucose	H183
31	0.09	1.008		
32	HGA2	1	bufalin-N-glucose	H121
32	0.09	1.008		
33	HGA2	1	bufalin-N-glucose	H122
33	0.09	1.008		
34	HGA2	1	bufalin-N-glucose	H111
34	0.09	1.008		
35	HGA2	1	bufalin-N-glucose	H112
35	0.09	1.008		
36	HGA1	1	bufalin-N-glucose	H9
36	0.09	1.008		
37	HAL3	1	bufalin-N-glucose	H191
37	0.09	1.008		
38	HAL3	1	bufalin-N-glucose	H192
38	0.09	1.008		
39	HAL3	1	bufalin-N-glucose	H193
39	0.09	1.008		
40	HGA2	1	bufalin-N-glucose	H11
40	0.09	1.008		
41	HGA2	1	bufalin-N-glucose	H12
41	0.09	1.008		
42	HGA2	1	bufalin-N-glucose	H21
42	0.09	1.008		
43	HGA2	1	bufalin-N-glucose	H22
43	0.09	1.008		
44	HGA1	1	bufalin-N-glucose	H3
44	0.09	1.008		
45	HGA2	1	bufalin-N-glucose	H41
45	0.09	1.008		
46	HGA2	1	bufalin-N-glucose	H42
46	0.09	1.008		
47	HGA1	1	bufalin-N-glucose	H5
47	0.09	1.008		
48	HGA2	1	bufalin-N-glucose	H61
48	0.09	1.008		
49	HGA2	1	bufalin-N-glucose	H62
49	0.09	1.008		
50	HGA2	1	bufalin-N-glucose	H71
50	0.09	1.008		
51	HGA2	1	bufalin-N-glucose	H72
51	0.09	1.008		

52	HGA1	1	bufalin-N-glucose	H8
52	0.09	1.008		
53	HOL	1	bufalin-N-glucose	HO14
53	0.42	1.008		
54	HGA2	1	bufalin-N-glucose	H151
54	0.09	1.008		
55	HGA2	1	bufalin-N-glucose	H152
55	0.09	1.008		
56	HGA2	1	bufalin-N-glucose	H161
56	0.09	1.008		
57	HGA2	1	bufalin-N-glucose	H162
57	0.09	1.008		
58	HGA1	1	bufalin-N-glucose	H17
58	0.09	1.008		
59	HGR62	1	bufalin-N-glucose	H2
59	0.268	1.008		
60	HGR62	1	bufalin-N-glucose	H23
60	0.199	1.008		
61	HGR62	1	bufalin-N-glucose	H1
61	0.284	1.008		
62	OG301	1	bufalin-N-glucose	O62
62	-0.17	15.999		
63	CG331	1	bufalin-N-glucose	C63
63	-0.1	12.011		
64	HGA3	1	bufalin-N-glucose	H64
64	0.09	1.008		
65	HGA3	1	bufalin-N-glucose	H65
65	0.09	1.008		
66	HGA3	1	bufalin-N-glucose	H66
66	0.09	1.008		
67	HCA1	1	bufalin-N-glucose	H67
67	0.09	1.008		
68	CC3163	1	bufalin-N-glucose	C68
68	0.11	12.011		
69	OC3C61	1	bufalin-N-glucose	O69
69	-0.4	15.999		
70	CC3162	1	bufalin-N-glucose	C70
70	0.27	12.011		
71	CC3161	1	bufalin-N-glucose	C71
71	0.14	12.011		
72	CC3161	1	bufalin-N-glucose	C72
72	0.14	12.011		
73	CC3161	1	bufalin-N-glucose	C73
73	0.14	12.011		
74	HCA1	1	bufalin-N-glucose	H74
74	0.09	1.008		
75	CC321	1	bufalin-N-glucose	C75
75	0.05	12.011		
76	OC311	1	bufalin-N-glucose	O76
76	-0.65	15.999		

77	HCA1	1	bufalin-N-glucose	H77
77	0.09	1.008		
78	HCA1	1	bufalin-N-glucose	H78
78	0.09	1.008		
79	HCA1	1	bufalin-N-glucose	H79
79	0.09	1.008		
80	OC311	1	bufalin-N-glucose	O80
80	-0.65	15.999		
81	HCA2	1	bufalin-N-glucose	H81
81	0.09	1.008		
82	OC311	1	bufalin-N-glucose	O82
82	-0.65	15.999		
83	HCA2	1	bufalin-N-glucose	H83
83	0.09	1.008		
84	HCP1	1	bufalin-N-glucose	H84
84	0.42	1.008		
85	HCP1	1	bufalin-N-glucose	H85
85	0.42	1.008		
86	HCP1	1	bufalin-N-glucose	H86
86	0.42	1.008		
87	OC311	1	bufalin-N-glucose	O87
87	-0.65	15.999		
88	HCP1	1	bufalin-N-glucose	H88
88	0.42	1.008		

#bufalin-N-glucose.prm

```
[ bondtypes ]
;    i      j  func         b0         kb
  CG2R57   CG3C51    1   0.15100000    292880.00
  CG2R57   CG3C52    1   0.15100000    292880.00
  CG3RC1   OG311    1   0.14200000    358150.40
  CC3261   CC3162    1   0.14800000    186188.00
   CG311   OC301    1   0.14150000    301248.00

[ angletypes ]
;    i      j      k  func     theta0      ktheta        ub0         kub
  CG2R53   CG2R51   CG2R57    5   116.500000   836.800000   0.00000000        0.00
  CG2R51   CG2R57   CG3C51    5   109.000000   962.320000   0.00000000        0.00
  CG2R51   CG2R57   CG3C52    5   109.000000   962.320000   0.00000000        0.00
  CG3C51   CG2R57   CG3C52    5   105.800000   753.120000   0.00000000        0.00
   CG311    CG311    OC311    5   109.700000   962.320000   0.00000000        0.00
   CG311    CG311   OG3C61    5   111.500000   376.560000   0.00000000        0.00
   CG321    CG311    OC311    5   109.700000   962.320000   0.00000000        0.00
   CG321    CG311    OC301    5   109.000000   376.560000   0.00000000        0.00
   CG321    CG311   OG3C61    5   111.500000   376.560000   0.00000000        0.00
   CG331    CG311   OG3C61    5   111.500000   376.560000   0.00000000        0.00
  OG3C61    CG311     HGA1    5   109.500000   376.560000   0.00000000        0.00
  CG2R57   CG3C51   CG3C52    5   106.000000   435.136000   0.00000000        0.00
  CG2R57   CG3C51   CG3RC1    5   106.000000   435.136000   0.00000000        0.00
  CG2R57   CG3C51     HGA1    5   112.600000   435.136000   0.00000000        0.00
  CG2R57   CG3C52   OG3C51    5   102.400000   627.600000   0.00000000        0.00
```

```
CG2R57  CG3C52   HGA2   5  112.600000  435.136000  0.00000000      0.00
CG311   CG3RC1   OG311  5  113.500000  488.272800  0.25610000   9338.69
CG3C52  CG3RC1   OG311  5  110.000000  633.457600  0.00000000      0.00
CG3RC1  CG3RC1   OG311  5  111.000000  446.432800  0.25610000   6694.40
CC3162  OC301    CG311  5  109.200000  418.400000  0.00000000      0.00
CG3RC1  OG311    HGP1   5  109.000000  418.400000  0.00000000      0.00
CG311   OG3C61   CG311  5  109.700000  794.960000  0.00000000      0.00
CC3261  CC3162   HCA1   5  110.100000  288.696000  0.21790000  18853.10
CC3261  CC3162   OC3C61 5  106.000000  376.560000  0.00000000      0.00
OC301   CG311    HGA1   5  109.500000  502.080000  0.00000000      0.00
CC3261  CC3162   OC311  5  107.000000  633.457600  0.00000000      0.00
OC301   CC3162   CC3261 5  105.000000  376.560000  0.00000000      0.00
CC3162  CC3261   CC3161 5  111.000000  446.432800  0.25610000   6694.40
CC3162  CC3261   HCA2   5  110.100000  288.696000  0.21790000  18853.10

[ dihedraltypes ]
;    i     j     k     l  func     phi0      kphi  mult
CG2R57  CG2R51  CG2R53  OG2D1   9  180.000000   5.020800  2
CG2R57  CG2R51  CG2R53  OG3C51  9  180.000000   1.715440  1
CG2R53  CG2R51  CG2R57  CG3C51  9  180.000000  27.614400  2
CG2R53  CG2R51  CG2R57  CG3C52  9  180.000000  27.614400  2
HGR51   CG2R51  CG2R57  CG3C51  9  180.000000  12.133600  2
HGR51   CG2R51  CG2R57  CG3C52  9  180.000000  12.133600  2
CG2R51  CG2R57  CG3C51  CG3C52  9  180.000000   0.209200  3
CG2R51  CG2R57  CG3C51  CG3RC1  9  180.000000   0.209200  3
CG2R51  CG2R57  CG3C51  HGA1    9    0.000000   0.000000  3
CG3C52  CG2R57  CG3C51  CG3C52  9    0.000000   9.916080  2
CG3C52  CG2R57  CG3C51  CG3RC1  9    0.000000   9.916080  2
CG3C52  CG2R57  CG3C51  HGA1    9  180.000000   0.000000  3
CG2R51  CG2R57  CG3C52  OG3C51  9  180.000000   1.171520  1
CG2R51  CG2R57  CG3C52  OG3C51  9  180.000000   4.100320  2
CG2R51  CG2R57  CG3C52  OG3C51  9  180.000000   7.322000  3
CG2R51  CG2R57  CG3C52  HGA2    9    0.000000   0.000000  3
CG3C51  CG2R57  CG3C52  OG3C51  9  180.000000   1.171520  1
CG3C51  CG2R57  CG3C52  OG3C51  9  180.000000   4.100320  2
CG3C51  CG2R57  CG3C52  OG3C51  9  180.000000   7.322000  3
CG3C51  CG2R57  CG3C52  HGA2    9  180.000000   0.000000  3
CG311   CG311   CG311   CG331   9  180.000000   2.092000  4
CG311   CG311   CG311   OG3C61  9  180.000000   0.794960  1
CG311   CG311   CG311   OG3C61  9  180.000000   4.184000  2
CG311   CG311   CG311   OG3C61  9    0.000000   2.510400  3
CG311   CG311   CG311   OG3C61  9  180.000000   0.334720  4
CG321   CG311   CG311   OG301   9  180.000000   0.836800  3
CG321   CG311   CG311   OC301   9  180.000000   0.836800  3
CG321   CG311   CG311   OG311   9    0.000000   0.585760  3
CG331   CG311   CG311   OG311   9    0.000000   0.585760  3
OG301   CG311   CG311   OG311   9    0.000000   0.836800  3
OG301   CG311   CG311   OG3C61  9    0.000000   0.836800  3
OC301   CG311   CG311   OG3C61  9    0.000000   0.836800  3
OG301   CG311   CG311   HGA1    9    0.000000   0.815880  3
OC301   CG311   CG311   HGA1    9    0.000000   0.815880  3
```

OG311	CG311	CG311	OG311	9	0.000000	0.836800	3
OG311	CG311	CG311	OG3C61	9	0.000000	0.836800	3
OG301	CG311	CG321	CG311	9	180.000000	0.669440	1
OG301	CG311	CG321	CG311	9	0.000000	1.631760	2
OC301	CG311	CG321	CG311	9	0.000000	0.836800	3
OC301	CG311	CG321	CG321	9	0.000000	0.836800	3
OG301	CG311	CG321	HGA2	9	0.000000	0.815880	3
OC301	CG311	CG321	HGA2	9	0.000000	0.815880	3
CG311	CG311	CG3RC1	OG311	9	0.000000	0.209200	3
CG321	CG311	CG3RC1	OG311	9	0.000000	0.209200	3
HGA1	CG311	CG3RC1	OG311	9	0.000000	0.209200	3
CG311	CG311	OG301	CG311	9	0.000000	1.673600	1
CG311	CG311	OG301	CG311	9	0.000000	2.050160	3
CG321	CG311	OC301	CC3162	9	180.000000	0.543920	1
CG321	CG311	OC301	CC3162	9	180.000000	1.046000	2
CG321	CG311	OC301	CC3162	9	180.000000	0.251040	3
HGA1	CG311	OG301	CG311	9	0.000000	1.188256	3
HGA1	CG311	OC301	CC3162	9	0.000000	1.188256	3
CG2R57	CG3C51	CG3C52	CG3C52	9	180.000000	15.480800	3
CG2R57	CG3C51	CG3C52	CG3C52	9	0.000000	9.414000	4
CG2R57	CG3C51	CG3C52	CG3C52	9	180.000000	0.962320	6
CG2R57	CG3C51	CG3C52	HGA2	9	0.000000	0.585760	3
CG2R57	CG3C51	CG3RC1	CG311	9	180.000000	15.480800	3
CG2R57	CG3C51	CG3RC1	CG311	9	0.000000	9.414000	4
CG2R57	CG3C51	CG3RC1	CG311	9	180.000000	0.962320	6
CG2R57	CG3C51	CG3RC1	CG331	9	180.000000	15.480800	3
CG2R57	CG3C51	CG3RC1	CG331	9	0.000000	9.414000	4
CG2R57	CG3C51	CG3RC1	CG331	9	180.000000	0.962320	6
CG2R57	CG3C51	CG3RC1	CG3RC1	9	0.000000	0.627600	3
CG3C52	CG3C52	CG3RC1	OG311	9	180.000000	2.092000	1
CG3C52	CG3C52	CG3RC1	OG311	9	0.000000	2.928800	2
CG3C52	CG3C52	CG3RC1	OG311	9	0.000000	1.673600	3
CG3C52	CG3C52	CG3RC1	OG311	9	0.000000	1.673600	5
HGA2	CG3C52	CG3RC1	OG311	9	180.000000	0.815880	3
CG2R57	CG3C52	OG3C51	CG2R53	9	180.000000	1.297040	1
CG2R57	CG3C52	OG3C51	CG2R53	9	0.000000	1.171520	6
CG311	CG3RC1	CG3RC1	OG311	9	0.000000	16.736000	3
CG331	CG3RC1	CG3RC1	OG311	9	0.000000	0.209200	3
CG3C51	CG3RC1	CG3RC1	OG311	9	0.000000	5.020800	3
CG311	CG3RC1	OG311	HGP1	9	0.000000	1.213360	1
CG311	CG3RC1	OG311	HGP1	9	0.000000	2.594080	2
CG311	CG3RC1	OG311	HGP1	9	0.000000	0.209200	3
CG3C52	CG3RC1	OG311	HGP1	9	0.000000	1.213360	1
CG3C52	CG3RC1	OG311	HGP1	9	0.000000	2.594080	2
CG3C52	CG3RC1	OG311	HGP1	9	0.000000	0.209200	3
CG3RC1	CG3RC1	OG311	HGP1	9	0.000000	6.276000	1
CG3RC1	CG3RC1	OG311	HGP1	9	180.000000	1.255200	2
CG3RC1	CG3RC1	OG311	HGP1	9	0.000000	1.338880	3
CG311	OC301	CC3162	OC3C61	9	180.000000	0.209200	1
CG311	OC301	CC3162	OC3C61	9	0.000000	3.807440	2
CG311	OC301	CC3162	OC3C61	9	180.000000	5.313680	3

CC3161	CC3261	CC3162	OC301	9	0.000000	0.836800	3
CC3261	CC3162	OC301	CG311	9	180.000000	1.715440	1
CC3261	CC3162	OC301	CG311	9	180.000000	2.761440	2
CC3261	CC3162	OC301	CG311	9	0.000000	6.694400	3
CG311	OC301	CC3162	HCA1	9	0.000000	1.188256	3
CC3161	CC3261	CC3162	OC3C61	9	180.000000	1.297040	3
CC3161	CC3162	OC3C61	CC3163	9	0.000000	0.836800	3
HCA1	CC3261	CC3162	OC3C61	9	0.000000	0.836800	3
CC3261	CC3162	OC3C61	CC3163	9	0.000000	0.836800	3
HCA2	CC3261	CC3162	OC3C61	9	0.000000	0.836800	3
HCA2	CC3261	CC3162	OC301	9	0.000000	0.836800	3
HCA1	CC3162	CC3261	CC3161	9	0.000000	0.836800	3
HCA1	CC3162	CC3261	HCA2	9	0.000000	0.836800	3
CC3261	CC3162	OC301	CC3161	9	180.000000	1.715440	1
CC3261	CC3162	OC301	CC3161	9	180.000000	2.761440	2
CC3261	CC3162	OC301	CC3161	9	0.000000	6.694400	3
CC3161	CC3161	CC3261	CC3162	9	180.000000	0.794960	3
HCA1	CC3161	CC3261	CC3162	9	0.000000	0.836800	3
CC3162	CC3261	CC3161	OC311	9	0.000000	0.836800	3
CC3261	CC3161	CC3161	OC301	9	0.000000	0.836800	3
CC3261	CC3161	CC3161	OC311	9	0.000000	0.836800	3

[dihedraltypes]
; 'improper' dihedrals
; i j k l func phi0 kphi
